# Survival after recurrence following surgical resected non-small cell lung cancer: A multicenter, prospective cohort study

**DOI:** 10.1016/j.xjon.2022.03.004

**Published:** 2022-04-04

**Authors:** Tomoyoshi Takenaka, Tokujiro Yano, Koji Yamazaki, Tatsuro Okamoto, Motoharu Hamatake, Mototsugu Shimokawa, Masaki Mori, Kyushu University Lung Surgery Study Group Japan

**Affiliations:** a Department of Surgery and Science, Graduate School of Medical Sciences, Kyushu University, Fukuoka, Japan; b Department of General Thoracic Surgery, Beppu Medical Center, National Hospital Organization, Beppu, Japan; c Department of Thoracic Surgery and Clinical Research Institute, Kyushu Medical Center, National Hospital Organization, Fukuoka, Japan; d Department of Thoracic Oncology, National Kyushu Cancer Center, Fukuoka, Japan; e Department of Thoracic Surgery, Kitakyushu Municipal Medical Center, Kitakyushu, Japan; f Department of Biostatistics, Yamaguchi University Graduate School of Medicine, Ube, Japan

**Keywords:** non–small cell lung cancer, surgical resection, recurrence, post recurrence survival, ALK, anaplastic lymphoma kinase, CT, computed tomography, ECOG, Eastern Cooperative Oncology Group, EGFR, epidermal growth factor receptor gene, EGFR-TKI, epidermal growth factor receptor tyrosine kinase inhibitor, ICI, immune checkpoint inhibitor, NSCLC, non–small cell lung cancer, OS, overall survival, PRS, post-recurrence survival, PS, performance status

## Abstract

**Objectives:**

The optimal treatment for recurrent non–small cell lung cancer (NSCLC) has not been standardized. In this prospective cohort study, we evaluated post-recurrence survival (PRS) after treatment of recurrent NSCLC and identified prognostic factors after recurrence.

**Methods:**

This multicenter prospective cohort study was conducted in 14 hospitals. The inclusion criteria for this study were patients with recurrence after radical resection for NSCLC. Information about the patient characteristics at recurrence, tumor-related variables, primary surgery, and treatment for recurrence was collected. After registration, follow-up data, such as treatment and survival outcomes, were obtained every 3 months.

**Results:**

From 2010 to 2015, 505 cases were enrolled, and 495 cases were analyzed. As initial treatment for recurrence, 263 patients (53%) received chemotherapy, 46 (9%) received chemoradiotherapy, 98 (20%) had definitive radiotherapy, 14 (3%) received palliative radiotherapy, and 31 (6%) underwent surgical resection. The remaining 43 patients (9%) received supportive care. The median PRS and 5-year survival rates for all cases were 30 months and 31.9%, respectively. The median PRS according to the initial treatment was as follows: supportive care, 8 months; palliative radiotherapy, 16 months; definitive radiotherapy, 30 months; chemotherapy, 31 months; chemoradiotherapy, 35 months; and surgery, not reached. A multivariate analysis showed that the age, gender, performance status, histology presence of symptoms, duration from primary surgery to recurrence, and number of recurrent foci were independent prognostic factors for PRS.

**Conclusions:**

The PRS of patients with recurrent NSCLC was different depending on the patient's background characteristics and initial treatment for recurrence.

## Graphical abstract

**Figure:**
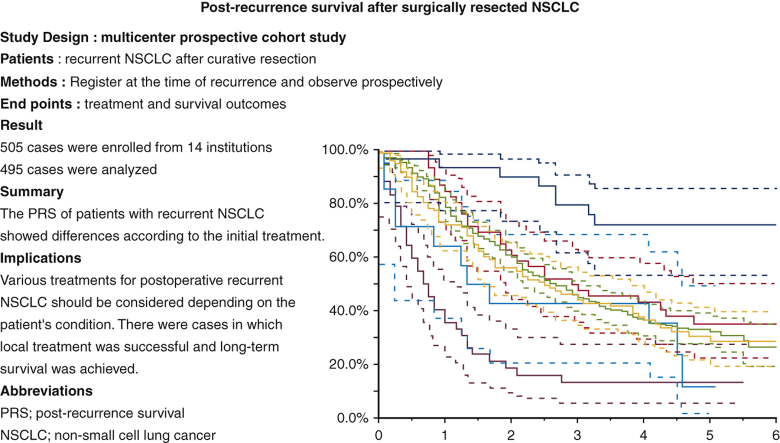

**Figure:**
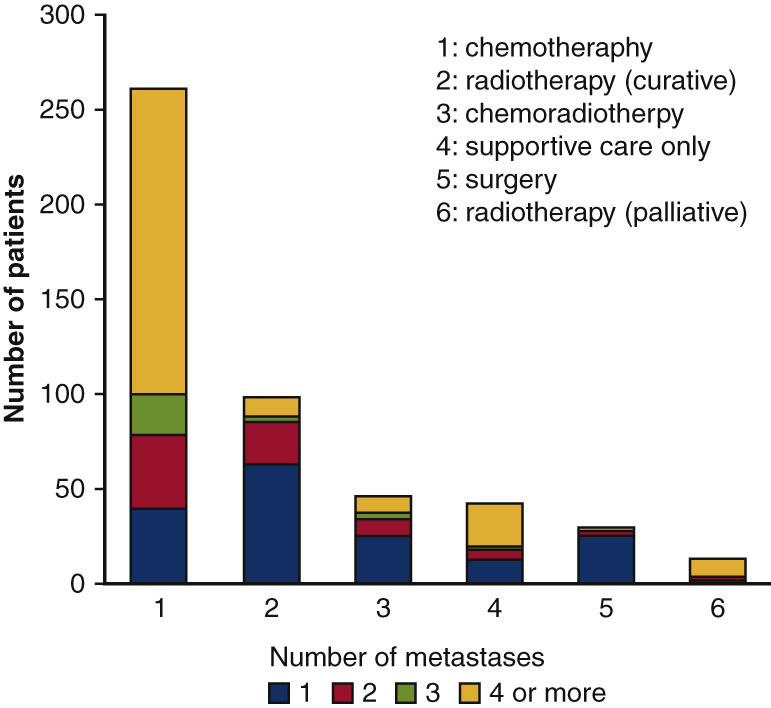
Correlation between number of recurrent foci and initial treatments.

**Central Message**

This prospective multicenter cohort study showed the outcomes of patients with recurrent NSCLC. Prognosis was very different because of the differences in patient characteristics and treatments.

**Perspective**

Considering the diversity of recurrent disease and the postoperative state, uniform treatment is unlikely to give the best treatment results for the patient with recurrent NSCLC. There were cases in which local treatment was successful and long-term survival was achieved in this study. More clinical studies will be needed to establish a treatment method according to the condition of recurrence.

See Commentary on page 382.

There were 2.0 million incident cases of lung cancer and 1.7 million deaths in 2016 in the world; lung cancer continues to be the most common type of cancer.^1^ Surgery is the best therapeutic modality for patients with early stage non–small cell lung cancer (NSCLC). Although the recurrence rate varies depending on the stage, it is reported to be 20% to 50%, even with recent advances in postoperative adjuvant chemotherapy.^2^, ^3^, ^4^, ^5^, ^6^, ^7^, ^8^

Treatment for recurrent disease is usually similar to that used for advanced diseases. According to the National Comprehensive Cancer Network guidelines for NSCLC, surgery and radiation therapy are recommended for resectable local recurrence, and concurrent chemoradiotherapy is recommended for mediastinal lymph node metastases.^9^ In contrast, systemic therapy is recommended for distant metastases, regardless of the recurrence organ (excluding the brain and bone) and number of recurrences.^9^ However, considering the diversity of recurrent disease and the postoperative state, controversy remains regarding whether the standard treatment for advanced disease should be the standard treatment for recurrent disease. According to the previous reports, local recurrence was reported to be 25% to 32% of all recurrent cases after resection of NSCLC.^2^, ^3^, ^4^, ^5^ Indeed, thoracic oligorecurrence of NSCLC has been reported to show a favorable outcome in a select population.^10^, ^11^, ^12^, ^13^, ^14^, ^15^, ^16^, ^17^, ^18^, ^19^ Although there is currently no clear consensus concerning the most appropriate treatment, local therapy, such as surgery, radiotherapy, or chemoradiotherapy, might be a common treatment option.^10^, ^11^, ^12^, ^13^, ^14^, ^15^, ^16^, ^17^, ^18^, ^19^ In addition, some patients who receive appropriate treatment might obtain a cure for recurrent disease.

Establishing future treatments for recurrent NSCLC requires collecting currently available real-world data concerning treatment results for recurrent NSCLC. Therefore, a prospective multicenter cohort study was designed to evaluate the post-recurrence survival (PRS) of patients with recurrent NSCLC to identify the prognostic factors associated with PRS and examine the outcome according to the initial treatment for recurrent disease.

## Methods

### Patients

The present trial, KLSS2, was planned by the Kyushu University Lung Surgery Study Group, started in July 2010, and was conducted in 14 hospitals in Japan. This study was a prospective observational study to evaluate the PRS after treatment of recurrent NSCLC, to evaluate the PRS according to the initial treatment for recurrent disease, and to identify the prognostic factors associated with PRS. The inclusion criteria for this study were patients with recurrence after radical resection for NSCLC. A total of 505 patients with recurrent NSCLC were enrolled in this study. This study was completed on March 31, 2021. Written informed consent was obtained from each patient according to the regulations of each participating institution. This study was approved by the institutional review board of Kyushu University Hospital (number 21-152; date approved: March 31, 2010).

### The Perioperative Examination

Preoperative diagnosis was determined on the basis of the findings of chest and upper abdomen computed tomography (CT) imaging, brain CT or magnetic resonance imaging, radionuclide bone scans, and/or fluorodeoxyglucose positron emission tomography. The histological diagnosis of the tumors was classified according to the World Health Organization classification of lung and pleural tumors in 1999 or the 2015 World Health Organization classification of tumors of the lung, pleura, thymus, and heart.^20^^,^^21^

**Figure E1:**
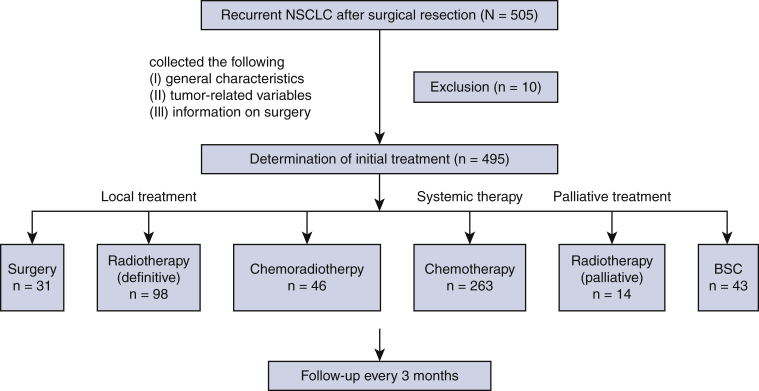
Consolidated Standards of Reporting Trials diagram of the study. *NSCLC*, Non–small cell lung cancer; *BSC*, best supportive care.

Postoperative follow-up examinations were performed according to the policy of each hospital. Generally, follow-up examinations are usually conducted every 3 to 4 months for the first 2 years and every 3 to 6 months thereafter. Routine follow-up procedures included physical examination, hematological examination, and chest radiography. In addition, chest and abdominal CT imaging was performed at least once per year. If recurrent disease was suspected, further evaluations, such as magnetic resonance imaging and fluorodeoxyglucose positron emission tomography, were also used. Recurrent NSCLC was diagnosed on the basis of findings consistent with recurrent disease on physical examination and diagnostic imaging. When clinically feasible, the diagnosis was histologically confirmed. The date of recurrence was defined as recurrence that was histologically proven or, in cases diagnosed on the basis of clinical evidence, when recurrent disease was recognized by the attending physician. The disease-free interval was defined as the period from the date of surgery to the date of recurrence detection. Local recurrence was defined as disease recurrence at the surgical margin, ipsilateral hemithorax, or mediastinum. Distant metastasis was defined as disease recurrence in the contralateral lung or outside the hemithorax and mediastinum. Local and distant metastases were defined as synchronous local and distant recurrence at diagnosis. A second lung tumor that was diagnosed as a different cell type from the first tumor before treatment was excluded. In cases in which there was no histological diagnosis, although solitary pulmonary nodules that met the Martini and Melamed^22^ criteria as a second primary lung cancer, such as a tumor without regional lymph node metastasis or distant metastasis that appeared more than 2 years after the first surgery, or nodules that the attending physician judged to be postoperative recurrence, were included in this study. The definition of oligo-recurrence in this study was cases with no more than 3 recurrent foci regardless of number of recurrent organs according to diagnostic imaging. The treatment policy for recurrence was also left to each hospital. The patient selection flow chart is shown in Figure E1.

### Data Collection and Extraction

For each patient, we collected the following information at the time of registration: (1) general characteristics at recurrence, (2) tumor-related variables, (3) information on surgery, and (4) treatment for recurrence. The general characteristics at recurrence included age, gender, Eastern Cooperative Oncology Group (ECOG) performance status (PS), symptoms of recurrence, physical findings, and recurrence confirmation date. Tumor-related variables included histological type, recurrent organs, recurrent site, number of recurrences, and epidermal growth factor receptor gene (*EGFR*) mutation status. Information on surgery included the date of surgery, side, type of resection, pathologic stage, and presence of postoperative adjuvant chemotherapy. Treatment for recurrence included the treatment for recurrence start date, initial treatment for recurrence, and chemotherapy regimen. After registration, follow-up data, such as treatment outcome, change of treatment, and survival outcome, were obtained every 3 months. Because the number of enrolled patients varied among the centers, to compare the treatment outcomes among the centers, the treatment outcomes were analyzed in 3 groups according to the number of enrolled patients: the first group consisted of the top 2 centers with the highest number of enrolled patients (high registration group), the second group consisted of the centers with the third to fifth highest number of enrolled patients (middle registration group), and the last group consisted of the remaining 9 centers (low registration group).

### Statistical Analyses

Comparisons of dichotomous variables between groups were performed using the χ^2^ test. The PRS was measured from the date of initial recurrence to the date of death from any cause or date on which the patient was last known to be alive. Survival probability was estimated using the Kaplan–Meier method. Differences in survival were evaluated using log rank tests. Univariate and multivariate analysis associated with PRS were tested using a Cox proportional hazards regression model. Analyses were performed using the JMP software package (version 11; SAS Institute Inc).

## Results

**Figure 1:**
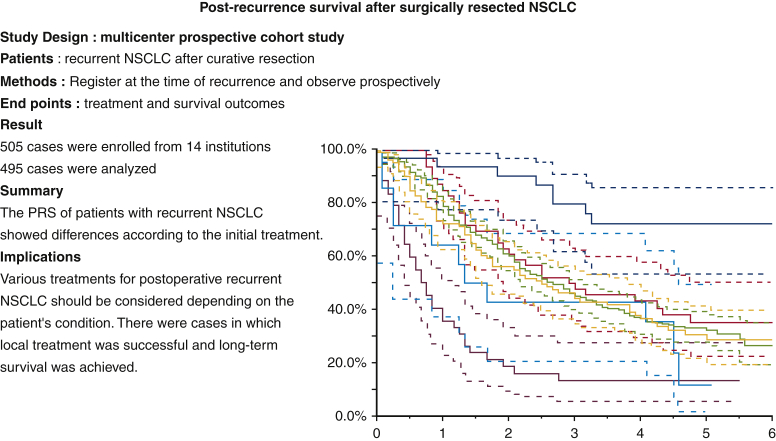
Study design and treatment outcomes. *NSCLC*, Non–small cell lung cancer; *PRS*, post-recurrence survival.

The characteristics of the patients at the time of recurrence are summarized in Table 1 and a summary of this study is shown in Figure 1. A total of 495 patients were analyzed in this study, including 322 men (65%) and 174 women (35%). The median age of the patients was 71 years (range, 32-98 years), and 445 (90%) had an ECOG PS of 0 or 1 at the time of initial recurrence. Adenocarcinoma accounted for 72% (n = 357) of the cases, followed by squamous cell carcinoma (n = 85; 17%) and other histological types (n = 53; 11%). Four hundred four patients underwent lobectomy, 25 underwent pneumonectomy, and 66 underwent sublobar resection as primary surgery. The median disease-free interval from surgery to the detection of the first recurrence was 14 months (range, 1-158 months). Among the 495 patients, 196 (40%) had local recurrence, 189 (38%) had distant recurrence, and 110 (22%) had local and distant recurrences. *EGFR* mutation status was assessed in 339 patients (68%), and 153 patients (31%) were positive for mutations. One hundred fifty-six patients were not tested for *EGFR* mutations. Sixty-eight cases had *EGFR* exon 19 deletions, 71 had exon21 L858R mutations, and 14 had other mutations. The most commonly involved organs were hilar and mediastinal lymph nodes in 211 cases, followed by the lung in 206, bone in 86, pleura in 60, brain in 53, pleura in 50, liver in 18, adrenal gland in 15, chest wall in 10, and other organs in 14 (Table 1). Relationship between the recurrence pattern and patients' characteristics are described in Table E1.

**Table 1:** Patient characteristics (N = 495)

Variable	Value
Age (range), y	71 (32-98)
Gender	
Male	322 (65)
Female	173 (35)
Performance status	
0	269 (54)
1	176 (36)
2, 3	50 (10)
Histological type	
Adenocarcinoma	357 (72)
Squamous cell carcinoma	85 (17)
Others	53 (11)
Primary operation	
Lobectomy	404 (82)
Pneumonectomy	25 (5)
Sublobar resection	66 (13)
Adjuvant chemotherapy	
Yes	234 (47)
No	261 (53)
Symptoms at recurrence	
With symptoms	140 (28)
Without symptoms	355 (72)
Recurrent site	
Distant recurrence	196 (40)
Local recurrence	189 (38)
Local and distant recurrence	110 (22)
Time to recurrence	
<1 Year	216 (44)
≥1 Year	279 (56)
Number of recurrent foci	
1	170 (34)
2	78 (16)
3	29 (6)
4 or more	218 (44)
Pathologic stage	
I	220 (44)
II	121 (24)
III	154 (31)
*EGFR* mutation status	
Positive	153 (31)
Negative	186 (38)
Unknown	156 (32)
Recurrent organs	
Hilar mediastinal lymph node	211 (42)
Supraclavicular and cervical lymph node	47 (9)
Intra-abdominal lymph node	16 (3)
Lung	206 (42)
Bone	86 (17)
Pleura	60 (12)
Brain	53 (11)
Liver	18 (3)
Adrenal grand	16 (3)
Chest wall	10 (2)
Other	14 (3)

Data are presented as n (%) except where otherwise indicated. *EGFR*, Epidermal growth factor receptor gene.

As an initial treatment for recurrent disease, chemotherapy was performed in 263 patients (53%), chemoradiotherapy was performed in 46 (9%), radiotherapy was performed in 112 (23%), and surgical resection was performed in 31 (6%). The remaining 43 patients (9%) received supportive care only (Table 2). Oligorecurrence more often received local treatment, such as surgery, chemoradiotherapy, and definitive radiotherapy, whereas distant recurrence, including the contralateral lung, more often underwent surgery (Table 2). Local recurrence more often received definitive radiotherapy and chemoradiotherapy (Table 2).

**Table 2:** Initial treatment according to recurrent number and recurrent pattern

Treatment	n (%)	Recurrent number (%)		Recurrent pattern, n (%)	
		Oligo recurrence	Multiple recurrence	Local recurrence	Distant recurrence
Supportive care only	43 (9)	19 (44)	24 (56)	21 (49)	22 (51)
Radiotherapy (palliative)	14 (3)	4 (29)	10 (71)	1 (7)	13 (93)
Radiotherapy (curative)	98 (20)	88 (90)	10 (10)	53 (53)	45 (47)
Chemotherapy	263 (53)	100 (38)	163 (62)	74 (28)	189 (72)
Chemoradiotherapy	46 (9)	37 (80)	9 (20)	35 (76)	11 (24)
Surgery	31 (6)	29 (94)	2 (6)	12 (39)	19 (61)

As for the treatment of organs with high recurrence frequency, treatment for hilar and mediastinal lymph node metastases cases (n = 211), chemotherapy was performed in 111 patients (53%), chemoradiotherapy was performed in 33 (16%), radiotherapy was performed in 40 (19%), and surgical resection was performed in 2 (1%), whereas 25 patients (12%) received supportive care. As an initial treatment for lung metastasis (n = 206), chemotherapy was performed in 127 patients (62%), chemoradiotherapy was performed in 7 (3%), radiotherapy was performed in 31 (15%), and surgical resection was performed in 25 (12%), whereas 16 patients (8%) received supportive care. There were 29 cases of solitary pulmonary nodules without regional lymph node metastasis or distant metastasis that appeared more than 2 years after the first surgery. Ten of the 29 cases were surgically resected and histologically diagnosed as recurrent lung cancer; the remaining cases were not histologically diagnosed and other treatments were chosen. As an initial treatment for bone metastasis (n = 86), chemotherapy was performed in 43 patients (49%), chemoradiotherapy was performed in 8 (9%), and radiotherapy was performed in 28 (33%), whereas 7 patients (8%) received supportive care.

The chemotherapy regimens for the chemotherapy group varied, but 154 patients were administered cytotoxic drugs, 104 patients were administered EGFR-tyrosine kinase inhibitors (EGFR-TKIs), and 5 patients were administered anaplastic lymphoma kinase (ALK) inhibitors as initial treatment for recurrent disease. None of the patients received immune checkpoint inhibitors (ICIs) for initial treatment. Chemotherapy regimens in the chemoradiotherapy group were 42 patients received cytotoxic drugs, and 4 patients received EGFR-TKIs.

**Figure 2:**
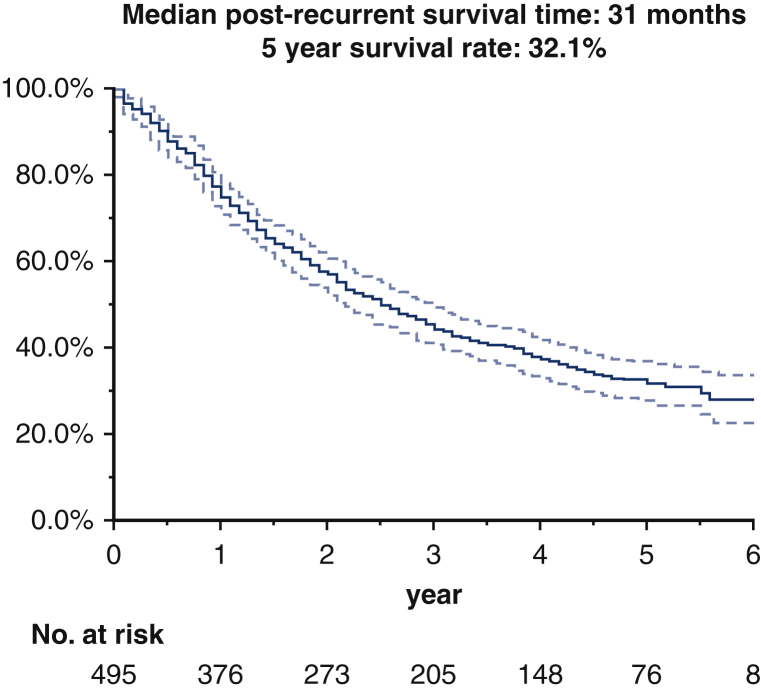
Kaplan–Meier curves of the post-recurrence survival of all cases. The *dotted line* represents the 95% confidence interval.

**Figure 3:**
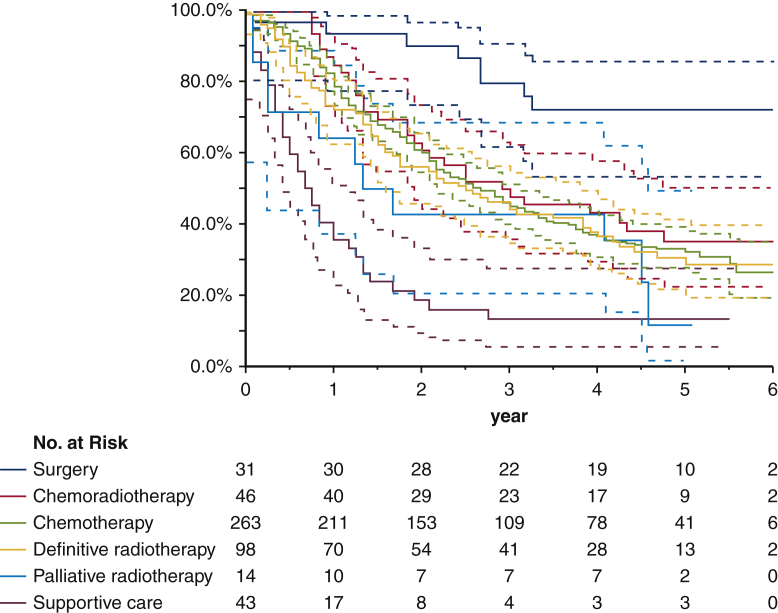
Kaplan–Meier curves of post-recurrence survival according to the initial treatments. *Blue line*: surgery; *red line*: chemoradiotherapy; *green line*: chemotherapy; *yellow line*: definitive radiotherapy; *cyan line*: palliative radiotherapy; *mauve line*: supportive care. The *dotted line* represents the 95% confidence interval.

**Figure E2:**
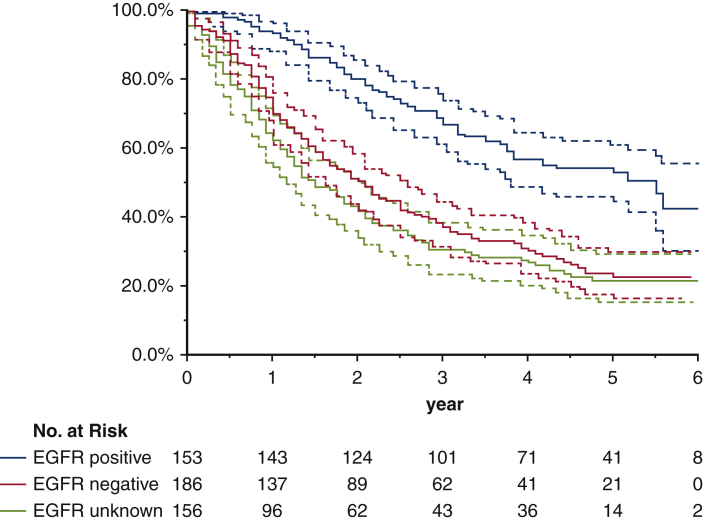
Kaplan–Meier curves of post-recurrence survival according to epidermal growth factor receptor gene (*EGFR*) mutation status.

The median follow-up period for survivors was 57 months (range, 1-89 months). The median PRS and 5-year survival rates of all patients were 30 months (range, 1-89 months) and 31.9%, respectively (Figure 2). The median PRS and 5-year PRS rates according to the initial treatment were as follows: supportive care, 8 months and 13.4%; palliative radiotherapy, 16 months and 11.9%; definitive radiotherapy, 30 months and 28.0%; chemotherapy, 31 months and 32.1%; chemoradiotherapy, 35 months and 35.2% and surgery, not reached, and 72.2%, respectively (Table 3 and Figure 3). Outcomes for local recurrence, distant recurrence, and oligorecurrence after initial treatment for recurrence are shown in Tables E2, E3, and E4. In all forms of recurrence, patients who underwent surgical resection had a good prognosis, whereas those who received only supportive care had a poor prognosis. Survival analysis according to *EGFR* mutation status is shown in Figure E2. The median PRS was 66 months for the patients with an *EGFR* mutation positive status, 25 months in the patients with a wild status, and 18 months in the patients with an unknown status (*P* < .001). Univariate analysis determined that age, sex, ECOG PS, histological type, presence of symptoms at recurrence, disease-free interval from initial surgery, pathologic stage, and number of recurrent foci influenced the PRS (Table 4). Multivariate analysis showed that age, sex, ECOG PS, histologic type, presence of symptoms at recurrence, disease-free interval from initial surgery, and number of recurrent foci influenced the PRS (Table 4). A summary of this study is also available in Video 1.

**Table 3:** Outcome for recurrent diseases after initial treatments

Treatment	n (%)	Median survival months (range)	HR (95% CI)	*P* value
Supportive care only	43 (9)	8 (1-66)	10.067 (4.912-23.383)	<.001
Radiotherapy (palliative)	14 (3)	16 (1-61)	4.913 (1.988-12.698)	
Radiotherapy (definitive)	98 (20)	30 (1-83)	3.707 (1.887-8.390)	
Chemotherapy	263 (53)	31 (1-89)	3.462 (1.822-7.673)	
Chemoradiotherapy	46 (9)	35 (9-74)	3.013 (1.444-7.070)	
Surgery	31 (6)	Not reached (1-77)	1.0 (–)	

*HR*, Hazard ratio; *CI*, confidence interval.

**Table 4:** Univariate and multivariate analyses of factors that predict post recurrence survival

	Univariate		Multivariate	
	HR (95% CI)	*P* value	HR (95% CI)	*P* value
Age				
≥ 75	1.581 (1.265-1.974)	<.001	1.615 (1.276-2.042)	<.001
75	1.0			
Gender				
Male	1.616 (1.275-2.063)	<.001	1.400 (1.081-1.823)	.011
Female	1.0			
Performance status				
≥2	3.782 (2.688-5.194)	<.001	3.350 (2.280-4.839)	<.001
0 or 1	1.0			
Histology				
Non-adenocarcinoma	1.860 (1.463-2.350)	<.001	1.635 (1.250-2.129)	<.001
Adenocarcinoma	1.0			
Primary operation				
Standard resection	1.078 (0.774-1.465)	.648	0.949 (0.670-1.370)	.773
Sublobar resection	1.0			
Adjuvant chemotherapy				
No	1.083 (0.869-1.352)	.477	1.134 (0.901-1.428)	.221
Yes	1.0			
Symptoms at recurrence				
With symptoms	2.346 (1.857-2.949)	<.001	1.672 (1.292-2.152)	<.001
Without symptoms	1.0			
Recurrent site				
Distant or both local and distant	1.154 (0.920-1.455)	.216	1.207 (0.952-1.536)	.121
Local	1.0			
Time to recurrence				
<1 Year	1.677 (1.345-2.091)	<.001	1.566 (1.242-1.973)	<.001
≥1 Year	1.0			
Number of recurrent foci				
≥4	1.854 (1.487-2.313)	<.001	2.063 (1.637-2.603)	<.001
1-3	1.0			
Pathologic stage				
≥ Stage II	1.286 (1.029-1.610)	.027	1.161 (0.915-1.480)	.221
Stage I	1.0			
Number of registrations per institutions				
Low registration institutions	1.236 (0.932-1.631)	.326	1.126 (0.817-1.379)	.715
High registration institutions	1.121 (0.867-1.451)		1.061 (0.817-1.379)	
Middle registration institutions	1.0			

*HR*, Hazard ratio; *CI*, confidence interval.

## Discussion

In this study, we prospectively evaluated the post-recurrence outcomes of patients with recurrence after curative resection for NSCLC. To our knowledge, there have been no reports of multicenter prospective observational studies of patients with recurrence after curative resection for NSCLC. Therefore, these results reflect the clinical outcomes of recurrent NSCLC. The median PRS time and 5-year survival rate of all patients were 30 (range, 1-89) months and 31.9%, respectively. In 2000, the median PRS of recurrent NSCLC was reported to be 17.7 to 25 months in Japan^2^, ^3^, ^4^; therefore, the prognosis of recurrent NSCLC seems to have improved over the past decade.

The effectiveness of local treatment in this study, such as surgery, definitive radiotherapy, and chemoradiotherapy, should be mentioned. According to the results, the PRS clearly varied according to treatment. Although the treatment method was not clearly defined for each recurrence pattern, definitive radiation therapy and chemoradiotherapy were often chosen for local recurrence. The results suggest that the prognosis after treatment for local lesions, such as with surgery or chemoradiotherapy, was better than that after systemic therapy or palliative therapy for systemic disease. The median survival time according to the initial local treatment for local recurrence was not reached for surgery, 52 months for chemoradiotherapy, and 21 months for definitive radiotherapy. Although locoregional recurrence is likely to cause troublesome symptoms, it is a potentially limited disease. Therefore, providing local control is important, and the administration of local treatment, such as radiotherapy or chemoradiotherapy, might be beneficial in cases of local recurrence after complete resection in patients without pleural dissemination or effusion. In particular, the results suggest that chemoradiotherapy might be more effective than radiation therapy alone for local recurrence.

Seol and colleagues^15^ evaluated the clinical outcomes of salvage radiotherapy for patients with lymph node recurrence that developed after radical surgery for NSCLC. They reported that the 1- and 2-year recurrence-free survival rates were 73.1% and 50.9%, respectively. Takenaka and colleagues^23^ reported the usefulness of concurrent chemoradiotherapy for recurrent NSCLC in select patients. They reported that 20% of patients who received concurrent chemoradiotherapy for local recurrence were disease-free for more than 3 years after chemoradiotherapy.

Oligorecurrence is usually defined as 3 to 5 or less distant metachronous metastases that can be treated with local therapy with controlled primary lesions.^10^, ^11^, ^12^, ^13^, ^14^, ^15^, ^16^, ^17^, ^18^, ^19^ Although the role of radical treatment for oligorecurrence is not well established, the subgroup of patients who received radical therapy, such as surgery or definitive radiotherapy, showed good PRS.^10^, ^11^, ^12^, ^13^, ^14^, ^15^, ^16^, ^17^, ^18^, ^19^ Indeed, a multivariate analysis showed that patients with oligorecurrence had a better PRS than those with multiple recurrences in this study. Han and colleagues^16^ reported the outcome of pulmonary oligorecurrence (5 or fewer metastatic lesions). They compared the PRS of patients who received operative or nonoperative treatment, including chemotherapy, radiotherapy, chemoradiotherapy, and best supportive care. The 5-year PRS rates in the operative and nonoperative groups were 67% and 26%, respectively. They concluded that operative treatment of pulmonary oligorecurrence significantly prolonged PRS in patients who underwent curative resection for NSCLC.^16^ Aoki and colleagues^17^ reported the outcome of salvage stereotactic body radiotherapy for oligorecurrence of NSCLC. The median overall survival (OS) after salvage stereotactic body radiotherapy was 32 months, and the 1- and 3-year OS rates were 84.4% and 67.8%, respectively. In the present study, 56% of the patients had oligorecurrence. Although local treatments, such as surgery, definitive radiotherapy, and chemoradiotherapy, were more often selected for oligorecurrence cases than for multiple recurrence cases, chemotherapy, palliative radiotherapy, and supportive care were selected for half of the oligorecurrence cases. In this study, 31 patients received surgery and 29 of them received surgery for oligorecurrence. Twenty-one patients received sublobar resection and 5 patients received lobectomy for lung metastasis. Two patients underwent tumor extirpation for brain metastasis, 1 patient received chest wall resection for chest wall recurrence, 1 patient received lymph node dissection, and 1 patient received small bowel resection. Local treatment for oligorecurrence might be expected to further improve prognosis.

The other reason for the improvement in the outcome of NSCLC recurrence is the development of chemotherapy, especially in molecular targeted drugs and immunotherapy. In this study, 112 patients were administered EGFR-TKIs as first-line therapy. In the 2010s, with the appearance of new drugs, such as molecular targeted therapy and immunotherapy, chemotherapy saw dramatic developments. In 2007, rearrangement of *ALK* was shown to be associated with the pathogenesis of a subset of patients with NSCLC.^24^ After the discovery of *ALK* rearrangement in lung cancer, the first-in-class ALK inhibitor crizotinib was shown to be superior to standard first-line chemotherapy in patients with previously untreated advanced *ALK*-positive NSCLC.^25^ Since then, several ALK inhibitors, such as alectinib and ceritinib, have been developed and shown to be superior to crizotinib.^26^^,^^27^ In this study, only 5 patients received ALK inhibitors as first-line treatment for recurrent disease. However, some patients were administered ALK inhibitors as second- or late-line therapy.

Recent advancements in cancer immunotherapies have revolutionized the treatment of advanced NSCLC by targeting immune checkpoints, such as programmed death-ligand 1 or its receptor, programmed cell death protein 1. By blocking the immune escape mechanism of the tumor, programmed death-ligand 1 or programmed cell death protein 1 inhibitors have been reported to have efficacy superior to conventional toxic chemotherapy, with a median OS exceeding 20 months in the first-line setting in some studies and a 1-year survival rate of nearly 70% in combination with platinum-based chemotherapy.^28^, ^29^, ^30^ Although none of the patients were administered ICIs for first-line treatment in this study, some patients who were treated after drug approval might benefit from second- or late-line ICI therapy. During the observation period, 35 patients received ICIs after second-line treatment in this study. Although not all patients received the latest chemotherapy in the present study, the median PRS of those who received chemotherapy as initial treatment for recurrence was 31 months, which was a satisfactory result.

This study had several limitations. First, this study was an observational study rather than an interventional one. The registration was not on the basis of strict regulations such as a histological or genetic diagnosis of recurrence. The fact that the diagnosis of recurrence was made according to the criteria of each institution cannot be denied. The treatment policy was also not shared among institutions. Because the diagnosis of recurrence, indications, and therapeutic strategies for recurrent disease were generally examined according to the standard of each institution, not all patients received treatment according to the same standard. Second, the PRS might be regulated not only by the initial therapy but also by the second- or third-line therapy. Indeed, some patients received new anticancer drugs, such as *ALK* inhibitors and ICIs, whereas others did not. Furthermore, because this was an observational study, the timing of treatment unavoidably had an effect. Despite these limitations, in this study we evaluated 495 patients with recurrent NSCLC who were prospectively registered at multiple institutions.

## Conclusions

The prognosis of postoperative recurrence of NSCLC has improved over the past decade. The PRS of patients with recurrent NSCLC differed depending on the patient's background characteristics and initial treatment. More clinical studies are needed to establish a treatment method on the basis of the condition of recurrence.

### Conflict of Interest Statement

The authors reported no conflicts of interest.

The *Journal* policy requires editors and reviewers to disclose conflicts of interest and to decline handling or reviewing manuscripts for which they may have a conflict of interest. The editors and reviewers of this article have no conflicts of interest.

## Figures and Tables

**Table E1:** Relationship of recurrence pattern to patient characteristics

Variable	Recurrence pattern, n (%)		*P* value
	Local recurrence	Distant recurrence	
Histological type			
Adenocarcinoma	120 (34)	237 (66)	.001
Squamous cell carcinoma	52 (61)	33 (39)	
Others	17 (32)	36 (68)	
Primary operation			
Lobectomy	142 (35)	262 (65)	.001
Pneumonectomy	5 (20)	20 (80)	
Sublobar resection	42 (64)	24 (36)	
Adjuvant chemotherapy			
Yes	86 (37)	148 (63)	.536
No	103 (39)	158 (71)	
Symptoms at recurrence			
With symptoms	42 (30)	98 (70)	.018
Without symptoms	147 (41)	208 (59)	
Time to recurrence			
<1 Year	76 (41)	140 (59)	.227
≥1 Year	113 (40)	166 (60)	
Pathologic stage			
Stage I	90 (41)	130 (59)	.361
Stage II	40 (33)	81 (67)	
Stage III	59 (38)	95 (62)	
*EGFR* mutation status			
Positive	49 (32)	104 (68)	.002
Negative	63 (34)	123 (66)	
Unknown	77 (49)	79 (51)	

*EGFR*, Epidermal growth factor receptor gene.

**Table E2:** Outcomes for local recurrence after initial treatments

Treatment	n (%)	Median survival months (range)	HR (95% CI)	*P* value
Supportive care only	21 (11)	10 (1-62)	6.857 (1.970-43.226)	<.001
Radiotherapy (palliative)	1 (1)	Not reached (-52)	Not evaluated	
Radiotherapy (definitive)	52 (27)	21 (2-63)	2.876 (0.877-17.714)	
Chemotherapy	74 (39)	37 (4-89)	1.723 (0.530-10.578)	
Chemoradiotherapy	35 (19)	52 (9-68)	1.536 (0.445-9.645)	
Surgery	6 (3)	Not reached (22-61)	–	

*HR*, Hazard ratio; *CI*, confidence interval.

**Table E3:** Outcomes for distant recurrence after initial treatments

Treatment	n (%)	Median survival months (range)	HR (95% CI)	*P* value
Supportive care only	22 (7)	5 (1-66)	11.447 (4.773-31.697)	<.001
Radiotherapy (palliative)	13 (4)	16 (1-61)	6.278 (2.385-18.245)	
Radiotherapy (definitive)	46 (15)	37 (1-83)	3.509 (1.556-4.248)	
Chemotherapy	189 (62)	29 (1-89)	4.308 (2.074-10.999)	
Chemoradiotherapy	11 (4)	15 (9-74)	7.178 (2.655-21.165)	
Surgery	25 (8)	Not reached (1-77)	–	

*HR*, Hazard ratio; *CI*, confidence interval.

**Table E4:** Outcome for oligo-recurrence after initial treatments

Treatment	n (%)	Median survival months (range)	HR (95% CI)	*P* value
Supportive care only	19 (7)	12 (2-66)	8.980 (3.585-25.454)	<.001
Radiotherapy (palliative)	4 (1)	51 (16-61)	3.766 (0.795-14.279)	
Radiotherapy (definitive)	88 (32)	33 (2-83)	4.462 (2.081-11.602)	
Chemotherapy	100 (36)	41 (1-89)	3.322 (1.549-8.634)	
Chemoradiotherapy	37 (13)	51 (9-74)	3.317 (1.422-9.039)	
Surgery	29 (10)	Not reached (11-77)	–	

*HR*, Hazard ratio; *CI*, confidence interval.

## Supplementary Data

Video 1 We present the study design, methods, and the results of the KLSS2 study. Video available at: https://www.jtcvs.org/article/S2666-2736(22)00130-9/fulltext.
