# Supplementary figures and images for: Survival after recurrence following surgical resected non-small cell lung cancer: A multicenter, prospective cohort study
Source: JTCVS Open. 2022 Apr 4;10:370–81. doi: 10.1016/j.xjon.2022.03.004 (PMC9390543; doi:10.1016/j.xjon.2022.03.004)

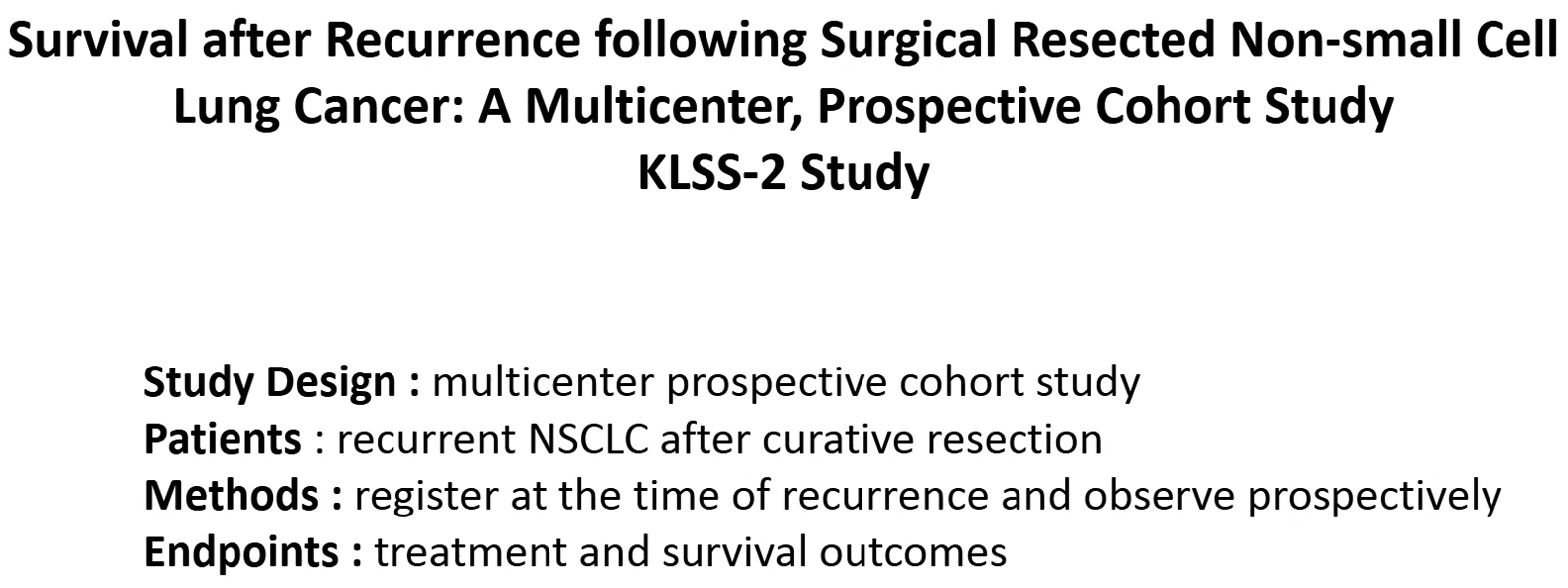

Supplement: Video 1 — We present the study design, methods, and the results of the KLSS2 study. Video available at: https://www.jtcvs.org/article/S2666-2736(22)00130-9/fulltext. [file fx3.jpg]
